# Interpenetrating Nanofibrous Composite Membranes for Removal and Reutilization of P (V) Ions from Wastewater

**DOI:** 10.3390/membranes15090262

**Published:** 2025-08-31

**Authors:** Guibin You, Hongyang Ma, Benjamin S. Hsiao

**Affiliations:** 1 State Key Laboratory of Organic-Inorganic Composites, Beijing University of Chemical Technology, Beijing 100029, China; 2 Department of Chemistry, Stony Brook University, Stony Brook, NY 11794-3400, USA; benjamin.hsiao@stonybrook.edu

**Keywords:** electrospinning, interpenetrating nanofibrous networks, lanthanum hydroxide, phosphate adsorption

## Abstract

Elevated phosphorus levels in wastewater created significant environmental concerns, including the degradation of surrounding soil structure, inhibition of plant growth, and potential threats to human health. To address this issue, a self-standing nanofibrous composite membrane based on PA-66/PVA-15%La(OH)_3_ was fabricated via electrospinning, followed by glutaraldehyde (GA) crosslinking and alkali hydrolysis to create an interpenetrating structure, where PA-66 provided the overall mechanical strength of the membrane, while La served as a functional component for the adsorption of phosphate. The chemical composition, surface morphology, thermal stability, and mechanical properties of the resulting membranes were characterized using ATR-FTIR, SEM, TGA, and tensile testing, respectively. Furthermore, the adsorption performance of the membranes was evaluated systematically through static and dynamic adsorption. The Langmuir isotherm model yielded a theoretical maximum adsorption capacity of 21.39 mg/g for phosphate ions. Notably, over 96% of this capacity was retained even in the presence of interfering ions. Moreover, dynamic adsorption experiments demonstrated that the membrane can deal with 1.74 L of phosphate-containing wastewater at a low flow rate of 1.0 mL/min and 1.46 L at a high flow rate of 2.0 mL/min, respectively, while consistently maintaining a phosphate removal efficiency exceeding 90%. A controlled release of phosphate ions from a phosphate-adsorbed membrane was successfully demonstrated using Mougeotia cultivation, implying the potential for phosphorus resource recovery.

## 1. Introduction

Water pollution, as a global environmental issue, involves phosphate ions, one of the primary contributors to eutrophication, which not only cause damage to aquatic ecosystems but also lead to economic losses in aquaculture, such as fish and shrimp farming, through cascading effects, thereby threatening ecosystem health and human socio-economic activities [1,2]. Consequently, the removal of excess phosphate from water bodies was one of the critical tasks in environmental protection. Currently, the removal of phosphate ions primarily relies on biological and chemical methods [3]. Biological phosphate removal typically exhibits a relatively slow rate and is susceptible to variations in water quality and quantity, temperature, dissolved oxygen, and other environmental factors, resulting in poor stability [4]. Chemical phosphorus removal, on the other hand, requires the addition of substantial amounts of chemical reagents (e.g., iron salts, aluminum salts, calcium salts, and magnesium salts), which may lead to secondary pollution. Furthermore, various anions present in water, such as SO_4_^2−^, CO_3_^2−^, and SiO_3_^2−^, may compete for adsorption sites with phosphate or participate in precipitation reactions, thereby hindering the selective removal of phosphorus [5]. Given that phosphorus is a non-renewable resource with limited global phosphate ore reserves, the recovery of phosphorus from wastewater is of vital strategic importance in terms of resources [6]. Therefore, achieving the resource utilization of phosphorus is also a crucial consideration in addressing phosphorus pollution problems.

Electrospun nanofibrous membranes have been widely applied in various fields, including air filtration [7], water filtration [8], food packaging [9], and electronic devices [10], due to their high porosity, large specific surface area, and tunable fiber diameter. As a result, they have also garnered significant interest for the treatment of water pollution caused by phosphate. PSF (polyethersulfone) nanofiber membranes [11] were prepared via electrospinning using DMF (dimethylformamide) and THF (tetrahydrofuran) as solvents and subsequently used for phosphate ion adsorption. Cerium (Ce) was coated onto the surface of electrospun PAN (polyacrylonitrile) nanofiber membranes, and phosphate adsorption experiments were conducted, achieving a maximum adsorption capacity of 17 mmol/g at pH = 2 [12]. Moreover, hematite (α-Fe_2_O_3_) and tetrabutylammonium bromide (TBAB) were incorporated into PAN, followed by electrospinning to fabricate PAN/Fe_2_O_3_/TBAB nanofiber membranes [13]. These membranes exhibited a maximum phosphate removal capacity of 8.76 mg/g at pH = 3. Another PAN-based membrane was prepared by functionalizing PAN electrospun nanofibers with akageneite (β-FeO(OH), denoted as Ak) and modifying them using the cationic surfactant benzyl dimethyl dodecyl chlorophenyl ammonium (BDDA) [14]. The influence of BDDA content on phosphate adsorption was investigated, and it was demonstrated that the phosphate adsorption capacity of the membrane increased linearly from 0.30 mmol P/g to 0.61 mmol P/g as the BDDA concentration was raised from 0% to 3%.

While significant progress has been made in utilizing electrospun nanofibrous membranes for phosphate adsorption, challenges such as limited selective adsorption capabilities and relatively low adsorption efficiencies still remain [15]. Lanthanum (La), known for its strong binding affinity with phosphate ions (with lanthanum phosphate having a low solubility product constant, pK_sp_ = 26.16) [16], has been explored as an adsorbent. La-loaded polyacrylonitrile (PAN) nanofiber membranes were fabricated via electrospinning combined with an in situ precipitation method, demonstrating excellent phosphate adsorption performance [17]. Meanwhile, polyvinyl alcohol/polyethylenimine (PVA/PEI) nanofiber membranes were also prepared through electrospinning and incorporated with La(NO_3_)_3_, achieving a high adsorption capacity of 165.9 mg P/g La in phosphate adsorption experiments [18]. However, it should be noted that incorporating inorganic phases can compromise the mechanical strength of the membrane. Additionally, conventional substrate attachment methods often suffer from insufficient adhesion strength between the substrate and the electrospun membrane [19]. To address these issues, PAN–PVA nanofibrous composite membranes were fabricated using a two-nozzle electrospinning technique and designed as a structure of interpenetrating nanofiber networks in which the crosslinked PVA component serves as a skeleton to provide mechanical properties, while the PAN component, after functionalization, is used for adsorption applications [20,21].

To address the issues of weak mechanical properties, poor adsorption selectivity, and low adsorption efficiency of traditional phosphate adsorption membranes, a composite nanofibrous membrane with an interpenetrating structure was fabricated using a two-nozzle electrospinning process. The spinning components were PA-66 and PVA loaded with lanthanum hydroxide, respectively, aiming to resolve the aforementioned three major problems. In the composite nanofibrous membrane, PA-66 primarily provided mechanical strength, while the PVA component mainly served as a carrier for La(OH)_3_, which possesses selective adsorption functionality. The membrane was then characterized comprehensively and applied for the adsorption of phosphate ions from wastewater, where adsorption dynamics, isotherms, thermodynamics, regeneration, and phosphate release were investigated systematically. The dynamic adsorption of phosphate ions demonstrated the membrane’s high adsorption capacity, while the subsequent phosphate release behavior offered a potential solution for the resource recovery and utilization of phosphate.

## 2. Materials and Methods

### 2.1. Reagents

Polyamide-66 (PA-66, M_w_ = 2.5 × 10^4^ g/mol) granules were purchased from Shanghai Rhawn, while formic acid (98%) and ascorbic acid were bought from Aladdin. Acetone and concentrated hydrochloric acid were from Beijing Fine Chemical Company, and sodium chloride and sodium sulfate came from Shanghai Test Agents. Other reagents such as potassium monobasic phosphate, potassium antimony tartrate, ammonium molybdate, polyvinyl alcohol (99.0–99.4 mol%, M_w_ = 7.3 × 10^5^ g/mol), and lanthanum chloride hexahydrate with a purity of 99.0% and glutaraldehyde (GA) were obtained from Shanghai Macklin.

### 2.2. Membrane Fabrication

The 16 wt% PA-66 spinning solution was prepared by dissolving a certain amount of PA-66 in absolute formic acid at 60 °C with continuous stirring for 2 h. The 12 wt% of PVA solution was prepared by blending PVA with lanthanum chloride under reflux at 120 °C for 48 h, with LaCl_3_ content varying from 0 to 15 wt%. Electrospinning was conducted using a two-nozzle device under the following conditions: applied voltage, 17.5 kV; needle inner diameter, 0.6 mm; nozzle-to-collector distance, 15 cm; solution flow rates, 0.98 μL/min (PA-66) and 20 μL/min (PVA); temperature, 26 °C; and humidity, 45%. Subsequently, the electrospun membrane was crosslinked by soaking in an acetone solution containing 30 mM GA and 0.01 mol HCl. The reaction was carried out at room temperature for 24 h, after which, the membrane was soaked in 0.1 mol/L sodium hydroxide solution for 30 min, followed by washing with deionized water until the washing solution reached a neutral pH (7.0). Finally, the membrane was dried under vacuum at 90 °C for 24 h.

### 2.3. Adsorption Experiments

#### 2.3.1. Static Adsorption of the Nanofibrous Composite Membranes

The adsorption capacity of the PA-66/PVA-15%La(OH)_3_ nanofibrous composite membrane was determined using phosphate ions as target adsorbates. The specific experimental protocol was as follows: 10 mg of the nanofibrous composite membrane was placed in a glass vial containing 10 mL of phosphate ion solution at a certain concentration. The effects of pH, adsorption dynamics, adsorption isotherms, adsorption thermodynamics, and interfering ion effects on adsorption were investigated. The concentration of the solution before and after adsorption was determined using a UV spectrophotometer at a wavelength of 710 nm; the concentration was determined using a cuvette with a path length of 1.3 mm. The colorimetric assay was performed using the molybdenum blue method [22].

#### 2.3.2. Dynamic Adsorption of the Nanofibrous Composite Membranes

The dynamic adsorption experiment was performed in a stainless-steel filtration cell with a diameter of 25 mm, connected to a peristaltic pump. In this experiment, the concentration of phosphate ions was 1.0 ppm, and the solution flow rate was set to 1.0 mL/min and 2.0 mL/min, respectively.

#### 2.3.3. Phosphorus Release of the Phosphate-Loaded Nanofibrous Nanofiber Membranes

To investigate whether the growth of Mougeotia was affected by the phosphorus-containing nanofibrous composite membrane and to evaluate the membrane’s release performance, the experimental procedure was as follows: approximately equal amounts of Mougeotia were placed in glass vials filled with 10 mL of deionized water and 10 mg of the membrane and incubated in the dark until the mass was reduced, indicating the consumption of stored nutrients. The release performance of the membrane was assessed by measuring the daily mass of the Mougeotia and monitoring the growth status of Mougeotia. The specific culture protocol is summarized in Table 1.

#### 2.3.4. Desorption and Regeneration Experiments

The recyclability of the nanofibrous composite membrane was evaluated through desorption and regeneration experiments. In the desorption experiment, the membrane after phosphate adsorption was first thoroughly washed with deionized water, followed by being immersed in a 0.5 mol/L NaOH solution. After desorption, the nanofibrous composite membrane was rinsed with deionized water, dried, and then subjected to the next adsorption cycle.

### 2.4. Characterizations

#### 2.4.1. Surface Morphologies and Pore Size

The surface morphology of the composite membrane was observed using a scanning electron microscope (SEM, S-4700, Hitachi, Tokyo, Japan), and 30–50 nanofibers were selected from the SEM image for fiber diameter analysis. To determine the membrane pore size, a pore size analyzer (JW-PD200) from Beijing Precision Gaobo Instrument (Beijing, China) was employed. Prior to the test, the membrane sample was soaked in GQ-16 infiltrating solution with a surface tension of 16 dynes/cm for 30 min.

#### 2.4.2. Mechanical Property Tests

The tensile test was conducted using a universal testing machine (Shenzhen Sansi UTM-5105, Shenzhen, China). The membrane sample was cut into a standard-size dumbbell-shaped specimen using a cutter mold. Three sets of parallel tests were conducted on the samples. The test was performed at a speed of 10 mm/min at room temperature, and the resulting stress–strain curve was plotted. To minimize measurement errors, the mean values for each group of three tests were calculated.

#### 2.4.3. Thermogravimetric Analysis

The thermogravimetric analyzer from Switzerland METTLER (model TGA/DSC3) was used to investigate the raw materials (PA-66, PVA, and LaCl_3_) and nanofibrous composite membranes with varying La(OH)_3_ contents at a heating rate of 10 °C/min over a temperature range of 30–800 °C, under a nitrogen atmosphere.

#### 2.4.4. Fourier Transform Infrared Spectroscopy

ATR-FTIR (IRTracer 100, Shimadzu Corporation, Kyoto, Japan) spectroscopy was employed to analyze the surface functional groups of the raw materials and the nanofibrous composite membranes before and after adsorption. All samples were dried in a vacuum oven at 60 °C for 24 h prior to testing.

#### 2.4.5. X-Ray Photoelectron Spectroscopy Measurements

X-ray photoelectron spectroscopy from Shimadzu Corporation, Japan (ESCALAB Xi+), was employed to analyze the elemental composition and surface molecular structure of the membranes. The experimental data were processed using advantage software and plotted with Origin 2019 software.

## 3. Results and Discussion

### 3.1. Characterization of Composite Nanofiber Membranes

#### 3.1.1. Surface Topography of the Nanofibrous Composite Membranes

**Figure 1:**
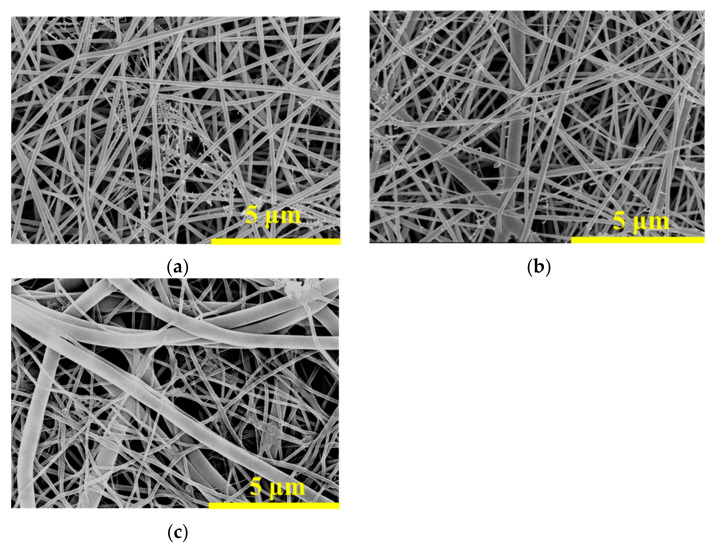
SEM images of (**a**) PA-66 nanofibrous membrane, (**b**) PA-66/PVA-5%La(OH)_3_ (5 wt% LaCl_3_ added) nanofibrous composite membrane after GA-crosslinking, and (**c**) PA-66/PVA-15%La(OH)_3_ (15 wt% LaCl_3_ added) nanofibrous composite membrane after GA-crosslinking.

The surface morphologies and composition of the nanofibrous composite membrane were observed using SEM, as shown in Figure 1.

The nanofibers in the PA-66 membrane are densely distributed, and the surface is smooth without obvious defects. The fine fibers attached to the main fibers are attributed to the ionization behavior of PA-66 in solution [23]. In Figure 1b, two types of nanofibers with different diameters can be clearly observed. Among them, the finer fibers are PA-66 fibers, while the thicker ones are PVA fibers, which are intertwined to form an interpenetrating network structure. With the increase in LaCl_3_ addition, the PVA fibers transition from straight to curved and from columnar to banded, as shown in Figure 1c. This morphological change is due to the uneven distribution of surface tension and internal stress caused by the evaporation of the solvent. The resulting inhomogeneity leads to varying degrees of fiber shrinkage and deformation during the curing process, ultimately resulting in fiber folding and collapse [24].

**Figure 2:**
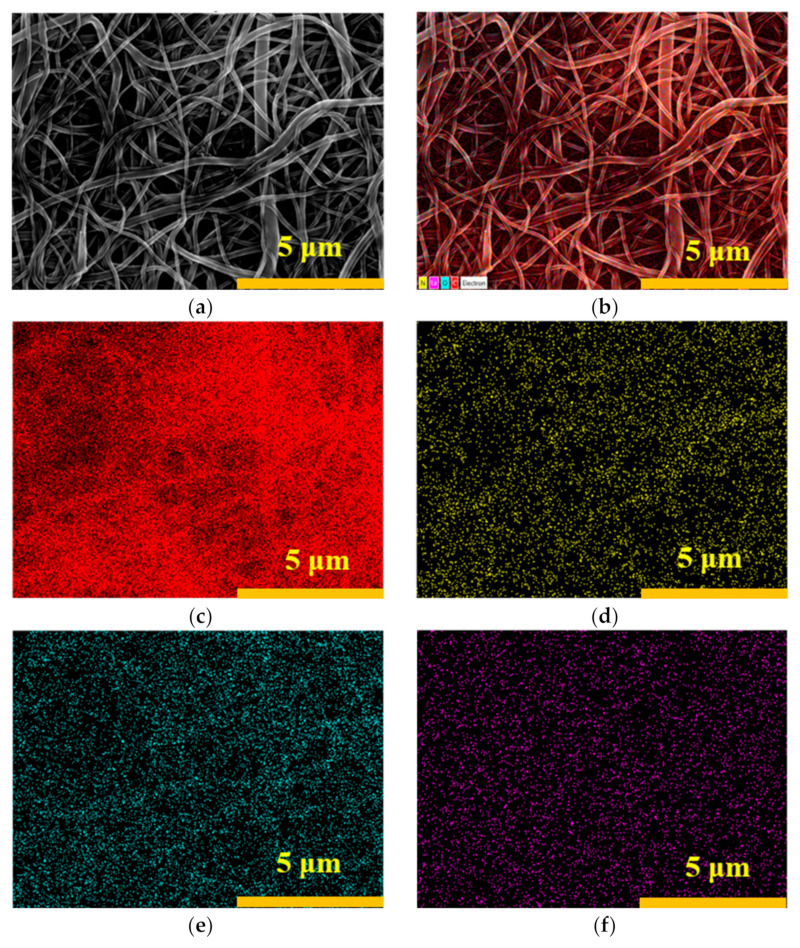
Original SEM image (**a**), EDS mapping image (**b**), elemental mappings of C (**c**), N (**d**), elemental mapping of O (**e**), and elemental mapping of La (**f**) for PA-66/PVA-15%La(OH)_3_.

As shown in Figure 2, the elemental distribution on the surface of the nanofibrous composite membrane is relatively uniform, indicating that La has been successfully incorporated into the membrane.

The measurements of fiber diameter and membrane pore size are presented in Table 2.

As shown in Table 2, the PA-66 fibers have a much finer diameter of only 0.110 μm and are not affected by the subsequent incorporation of LaCl_3_. In contrast, the PVA fibers are coarser, with a diameter of 0.558 μm when the LaCl_3_ content is only 5%, and this diameter increases with the addition of LaCl_3_. This trend is consistent with the SEM images of the fibers shown in Figure 1, indicating that during the spinning process, the two components are spun independently of each other. The nanofibrous composite membrane exhibits a porosity of 86.4%, as measured.

With the addition of lanthanum chloride, the average pore size of the membrane also increases. This is because LaCl_3_ significantly enhances the electrical conductivity of the solution, thereby increasing the charge carried by the jet. As a result, the electrostatic repulsion between the fibers is intensified. This increased electrostatic repulsion causes the fibers to deposit more loosely during the deposition process, forming a more relaxed fiber network and, consequently, larger pores.

After GA crosslinking, the pore sizes of the membranes are reduced to a certain extent. This reduction is attributed to the reaction of GA with the hydroxyl groups in PVA molecules, which leads to the formation of covalent bonds. These bonds make the molecular chains within the fibers more compact. The contraction of the molecular chains results in a decrease in the diameter of individual fibers, as well as an overall shrinkage of the fiber network, which collectively contributes to the reduction in membrane pore size.

In addition, GA forms crosslinking bridges between adjacent PVA fibers, bringing them closer together and tightening the fiber network, which further reduces the membrane pore size.

#### 3.1.2. Mechanical Properties

**Figure 3:**
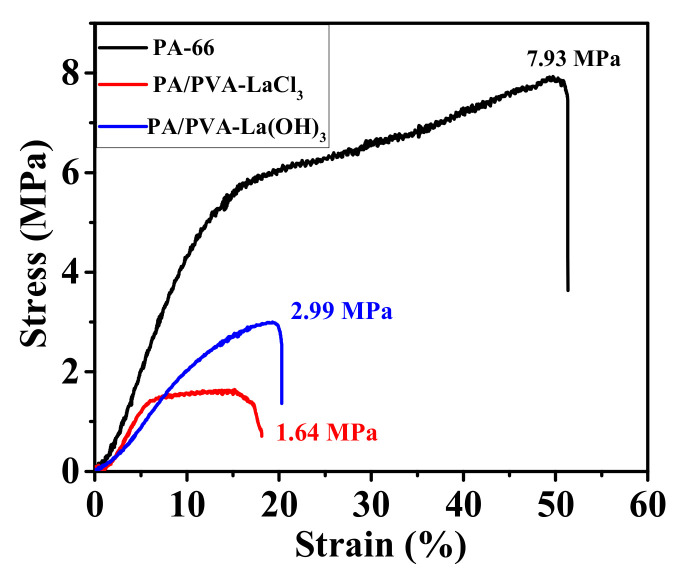
Stress–strain curves of nanofibrous membranes including PA-66, PA-66/PVA-LaCl_3_, and GA-crosslinked PA-66/PVA-15%La(OH)_3_.

The mechanical properties of PA-66, PA-66/PVA-LaCl_3_, and GA-crosslinked PA-66/PVA-15%La(OH)_3_ nanofibrous membranes were measured by tensile experiments, as shown in Figure 3.

From the stress–strain curves, it can be clearly observed that the tensile behavior of the PA-66 and PA-66/PVA-LaCl_3_ membranes before crosslinking is more similar. The PA-66 nanofibrous membrane exhibited an elastic modulus of 52.06 MPa. This similarity indicates that the membrane undergoes a transition from elastic to plastic deformation under stress, which is reflected on the curve as a sudden decrease in the slope. After crosslinking, the yield point of the composite nanofiber membrane PA-66/PVA-15%La(OH)_3_ disappears [25], and the slope becomes approximately the same as that of the initial elastic deformation stage observed before crosslinking. This suggests that the microscopic network structure of the nanofibrous composite membrane is fixed by GA, resulting in deformation that is predominantly elastic.

Based on the data, the tensile strength of the nanofibrous composite membrane increased from 1.64 ± 0.20 MPa to 2.99 ± 0.15 MPa after crosslinking, while the elastic modulus decreased from 33.68 ± 1.2 MPa to 25.19 ± 1.8 MPa. In contrast, the elongation at break increased slightly from 19.80% to 20.34%. These results indicate that crosslinking has enhanced both the yield strength and toughness of the composite membrane, thereby potentially extending its service life.

#### 3.1.3. Thermal Behavior

**Figure 4:**
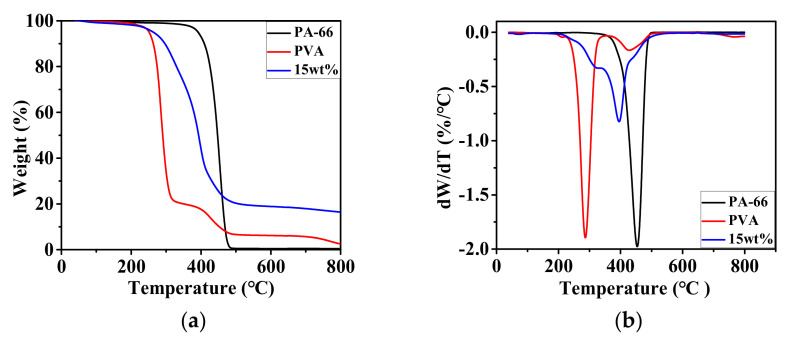
TGA/DTG curves of PA-66, PVA, and nanofibrous composite membranes: (**a**) TGA and (**b**) DTG, respectively.

The thermogravimetric curves of PA-66, PVA, and PA-66/PVA-15%La(OH)_3_ membranes were investigated, as shown in Figure 4.

As shown in Figure 4a, the decomposition of the PA-66 sample occurs in three stages [26]. In the first stage from 0 to 324 °C, the sample exhibits a weight loss of only 1.32%, which is attributed to the evaporation of residual moisture. The second stage, ranging from 324 to 484 °C, is a rapid weight loss phase where the sample’s mass is reduced to 0.81%, indicating thermal decomposition characterized by chain scission and the rupture of intramolecular amide bonds, releasing small molecule gases and other volatile organic compounds. The decomposition rate of PA-66 reaches its maximum at 454 °C (Figure 4b). In the third stage from 484 to 800 °C, the sample decomposes slowly, with a final residue of 0.33%.

The decomposition of the PVA sample occurs in four stages: 0–189 °C, 189–319 °C, 319–513 °C, and 513–800 °C [27], respectively. The slight weight loss in the first stage is due to the evaporation of water as PVA molecules contain a large number of hydroxyl groups that readily absorb moisture, with a weight loss of 0.92%. The second stage is a rapid weight loss phase where PVA undergoes dehydration, removing hydroxyl groups, followed by the scission of polymer chains to form small molecule gases. This stage is the primary decomposition phase of the sample, reaching its maximum decomposition rate at 287 °C. In the third stage, the weight loss rate decreases due to the thermal crosslinking of PVA fibers at high temperatures, which enhances their stability and leads to a reduced weight loss rate. The fourth stage involves the carbonization of the polymer chains, with a final residue of 2.41%.

The nanofibrous composite membrane exhibits weight loss behavior that is roughly similar to that of PA-66. However, due to the presence of PVA and La(OH)_3_ components, the rapid weight loss stage is stabilized within the temperature range of 218–484 °C [28].

#### 3.1.4. ATR-FTIR Measurements

**Figure 5:**
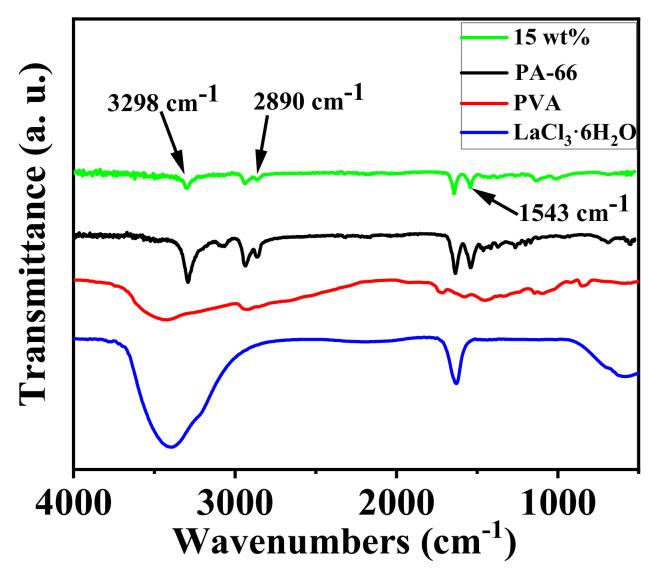
ATR-FTIR spectra of composite nanofibrous membranes and raw materials.

ATR-FTIR spectra of PA-66, PVA, LaCl_3_·6H_2_O, and nanofibrous composite membrane PA-66/PVA-15%La(OH)_3_ were achieved as exhibited in Figure 5.

By analyzing Figure 5, it can be observed that the composite membrane exhibits a single peak at 3298 cm^−1^, double peaks at 2890 cm^−1^ and 1543 cm^−1^, and a single peak at 1137 cm^−1^ [29,30,31]. Comparing the composite membrane’s spectrum with that of the raw materials, it is determined that the single peak at 3298 cm^−1^ corresponds to the O–H stretching vibration, the double peaks at 2890 cm^−1^ are attributed to the C–H stretching vibrations, and the double peaks at 1543 cm^−1^ are associated with the coupled vibrations of C–N and N–H stretching. The aldehyde group stretching vibration peak at 1137 cm^−1^ further confirms the successful GA crosslinking [32].

### 3.2. Adsorption Experiments of the Nanofibrous Composite Membrane

#### 3.2.1. Static Adsorption of the Nanofibrous Composite Membrane

**Figure 6:**
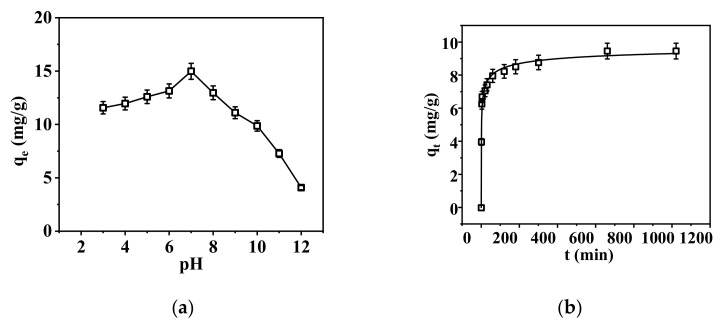
The influence of different factors on adsorption: (**a**) effect of pH and (**b**) effect of time on adsorption, respectively.

Based on the experimental results, the standard curve for P concentration is y = 0.505x − 0.0043 with R^2^ = 0.999. The effects of solution pH values and adsorption time were determined, respectively, as shown in Figure 6.

The effect of pH on phosphate adsorption was investigated, as shown in Figure 6a. The adsorption capacity of the nanofibrous composite membrane for phosphate first increased and then decreased with increasing pH, reaching a maximum adsorption capacity at pH = 6, with q_e_ = 14.98 mg/g. This is because pH affects the speciation of phosphate ions [33]. Under acidic conditions, PO_4_^3−^ mainly exists in the form of H_3_PO_4_, and as the pH of the solution increases, it gradually dissociates into H_2_PO_4_^−^, HPO_4_^2−^, and PO_4_^3−^. On one hand, the adsorption effect of the nanofibrous composite membrane on phosphate is influenced by the charge carried by the ions. According to the electrostatic attraction mechanism, the more charge an ion carries on its surface, the more easily it can be adsorbed [34]. From this perspective, it can be concluded that the higher the pH of the solution, the higher the content of PO_4_^3−^ in the solution and the higher the adsorption capacity of the membrane. However, on the other hand, the adsorption capacity of the membrane for phosphate is also affected by the competitive ions OH- in the solution. As the pH of the solution increases, the content of OH- in the solution also increases. Therefore, as the pH increases, the adsorption capacity of the membrane for phosphate decreases [35]. In summary, the adsorption of phosphate by the nanofibrous composite membrane shows a trend of first increasing and then decreasing due to the combined influence of the above two factors, and it exhibits the best adsorption performance at pH = 6.

**Figure 7:**
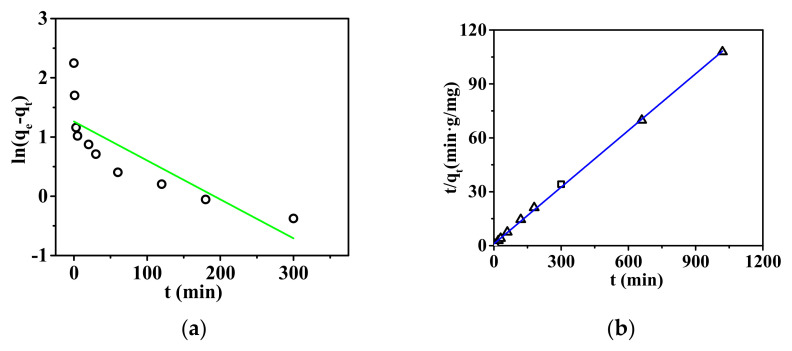
Adsorption kinetics fitting curve: (**a**) pseudo-first-order adsorption and (**b**) pseudo-second-order adsorption.

The adsorption capacity vs. adsorption time, i.e., adsorption kinetics, was determined, as shown in Figure 7.

As shown in Figure 7, the adsorption equilibrium time for phosphate ions by the nanofibrous composite membrane is 120 min. From Table 3, the kinetic data of the adsorption reaction were fitted using the pseudo-first-order and pseudo-second-order kinetic equations. The adsorption results of the nanofibrous composite membrane for phosphate ions are more consistent with the pseudo-second-order kinetic model, indicating that the adsorption process is not a simple physical adsorption but involves a more complex chemical adsorption mechanism [36]. This may include multiple steps such as the diffusion, adsorption, and desorption of phosphate ions on the membrane surface [37].

**Figure 8:**
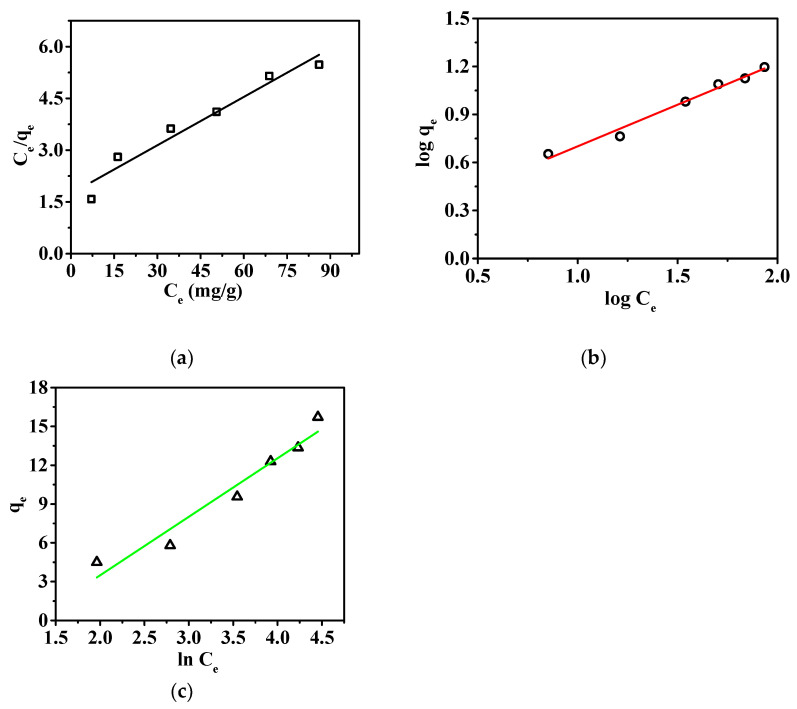
Adsorption isotherms: (**a**) Langmuir, (**b**) Freundlich, and (**c**) Tempkin adsorption isotherms, respectively.

Among the three isotherms, the Freundlich model provided the best fit, with R^2^ value of 0.98, as shown in Figure 8 and Table 4. This indicates that the surface of the membrane is heterogeneous, with different adsorption sites exhibiting varying adsorption capacities [38]. The adsorption is multilayered, and physical adsorption plays a significant role in the process. This observation differs slightly from the results obtained from adsorption kinetics; however, kinetics, as a direct description of the rate-controlling steps of the adsorption process, can better reflect the adsorption mechanism. The adsorption isotherm only reflects the behavior at adsorption equilibrium and cannot directly explain the dynamic characteristics of the adsorption process [39]. Additionally, the Tempkin model also supports the existence of chemical interactions between the nanofibrous composite membrane and the adsorbed ions [40]. Therefore, it can be concluded that the adsorption process of the nanofibrous composite membrane for phosphate ions is dominated by chemical adsorption, while physical adsorption also plays a certain role. Through the Langmuir adsorption model, the maximum adsorption capacity of the nanofibrous composite membrane for phosphate ions was determined to be 21.39 mg/g.

By comparing different adsorbents and La-based adsorbents, it was found that La exhibits superior adsorption efficiency for phosphate ions compared to conventional adsorbents. The specific data are presented in Table 5.

The data presented in the table indicate that the adsorption capacity of the composite nanofiber membrane is enhanced compared to pure La_2_O_3_-based adsorbents. This improvement is attributed to the high specific surface area of the composite nanofiber membrane, measured by BET as 5.55 m^2^/g. Furthermore, in comparison with conventional Mg-Al-based adsorbents, the PA-66/PVA-15%La(OH)_3_ composite exhibits higher adsorption selectivity for phosphate ions. This is attributed to the strong coordination effect between La and phosphate ions.

**Figure 9:**
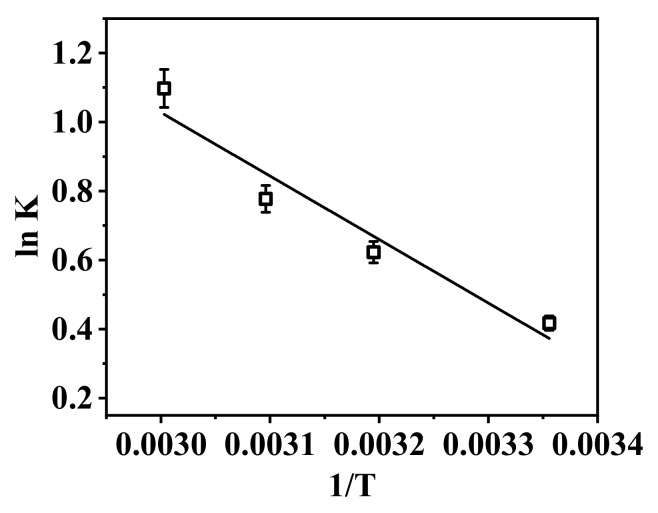
Thermodynamic adsorption of the nanofibrous composite membrane.

The thermodynamic adsorption behavior of the nanofibrous composite membrane PA-66/PVA-15%La(OH)_3_ was investigated, as shown in Figure 9 and Table 6.

From Figure 9, the fitting degree of the thermodynamic curve is reasonably high, with an R^2^ value of 0.983, and the thermodynamic parameters presented in Table 6 can be derived. It is evident that all ΔG values for the adsorption are negative, indicating that the adsorption of phosphate ions by the nanofibrous composite membrane is a spontaneous process [50]. Moreover, as the temperature increases, the spontaneity of the reaction also increases. On the other hand, the positive ΔH^0^ values suggest that the adsorption process is endothermic. With the increase in temperature, the adsorption equilibrium shifts to the right, which is more favorable for the adsorption process [51].

**Figure 10:**
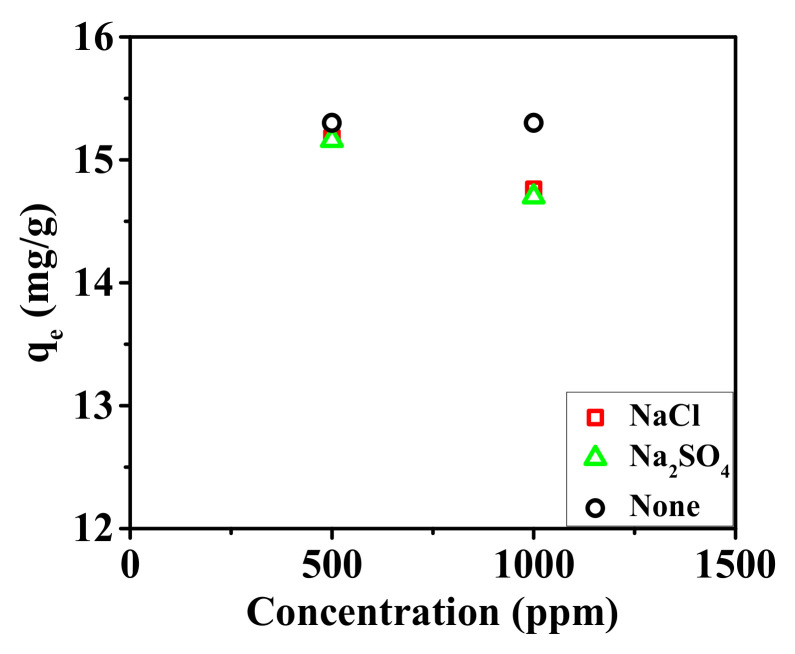
Effect of interfering ions on the adsorption of phosphate ions in the PA-66/PVA-15%La(OH)_3_ membrane.

The effects of the competing ions on the adsorption of the nanofibrous composite membrane were also investigated, as shown in Figure 10.

In an environment containing interfering ions, the adsorption capacity of the nanofibrous composite membrane for phosphate ions slightly decreases. The presence of 500 ppm NaCl and Na_2_SO_4_, as well as 1000 ppm NaCl and Na_2_SO_4_, results in a reduction in the adsorption capacity of the membrane by 0.78%, 0.91%, 3.53%, and 3.92%, respectively. It can be concluded that the adsorption of phosphate ions by the nanofibrous composite membrane is negligibly affected by interfering ions in the environment [52]. Therefore, overall, the nanofibrous composite membrane demonstrates a strong selective adsorption capability for phosphate ions.

#### 3.2.2. Dynamic Adsorption of the Nanofibrous Composite Membrane

**Figure 11:**
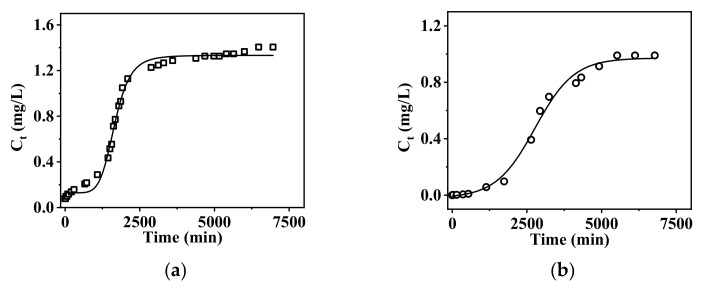
The dynamic adsorption breakthrough curves of PA-66/PVA-15%La(OH)_3_ membranes: (**a**) at low flow rate of 1.0 mL/min and (**b**) at high flow rate of 2.0 mL/min, respectively.

The dynamic adsorption of the membrane for phosphate ions was conducted, and the results are as shown in Figure 11.

From Figure 11, it can be observed that as the volume of wastewater treated increases, the concentration of phosphate ions in the effluent gradually rises, eventually leading to the loss of adsorption capacity. At a high flow rate of 2.0 mL/min, the membrane begins to gradually lose its adsorption capacity at 730 min and completely loses it by 2890 min. Based on this flow rate, it is estimated that a total of 5.78 L of wastewater was treated, with the first 1.46 L of effluent containing a phosphate concentration that is only 9.67% of the initial concentration. At the low flow rate of 0.2 mL/min, the membrane maintains 90% of its treatment capacity for up to 1740 min, and its adsorption capacity gradually decreases by 4920 min. Calculations indicate that it can efficiently treat 1.74 L of wastewater under these conditions.

**Figure 12:**
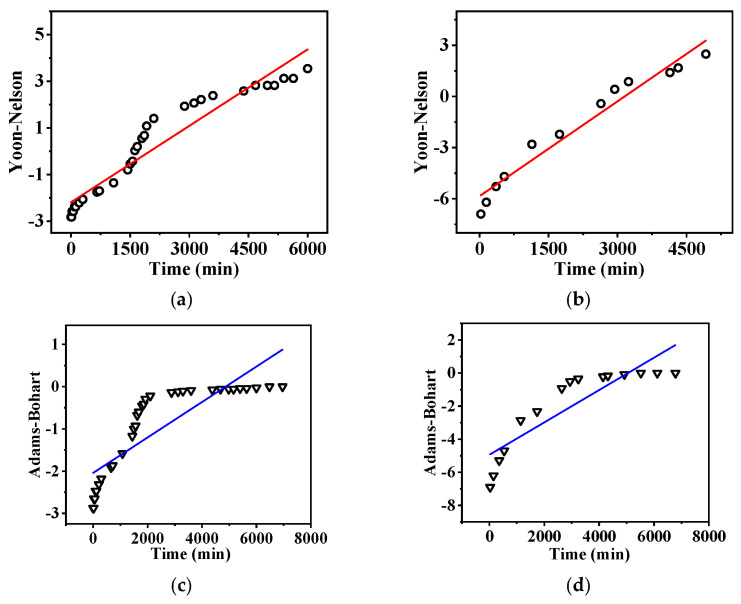
Dynamic adsorption fitting curves with different models at different flow rates: (**a**,**c**) at 1.0 mL/min and (**b**,**d**) at 2.0 mL/min, respectively.

Two models, Yoon-Nelson and Adamas-Bohart, are employed to simulate the dynamic adsorption of the nanofibrous composite membrane for phosphate ions, as shown in Figure 12.

From Table 7, it can be seen that the Yoon–Nelson model [53,54] provides a good fit for the dynamic adsorption of phosphate ions, indicating that the adsorption rate of the nanofibrous composite membrane is relatively fast. Furthermore, the 50% breakthrough time calculated using the Yoon–Nelson model is in excellent agreement with the experimental results. This suggests that the dynamic adsorption process of phosphate is well described by the Yoon–Nelson model.

By comparing the adsorption rate constants under low and high flow rates, it is evident that the rate constant for phosphate dynamic adsorption increases as the flow rate decreases. This suggests the formation of strong chemical bonds between phosphate ions and the adsorbent surface. Lower flow rates are more favorable for the formation and stabilization of these bonds [55], thereby enhancing the adsorption rate constant.

### 3.3. Adsorption Mechanism

**Figure 13:**
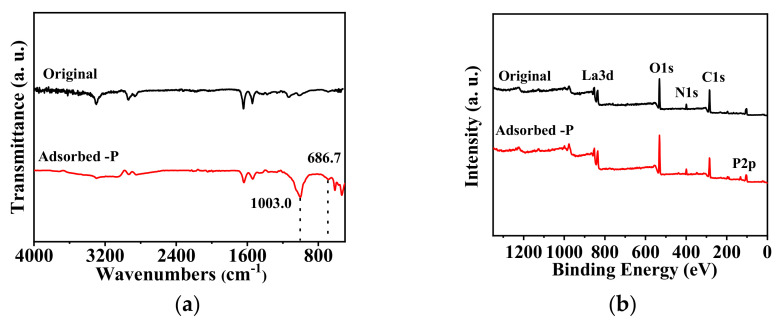
ATR- FTIR spectra of PA-66/PVA-15%La(OH)_3_ membranes before and after adsorption (**a**) and broad-spectrum of XPS before and after phosphate absorption (**b**).

The adsorption mechanism of the nanofibrous membrane for phosphate ions are explored and proposed based on ATR-FTIR and XPS measurements, as shown in Figure 13.

After adsorbing phosphate, the nanofibrous composite membrane exhibited a P-O stretching vibration peak at 1003.0 cm^−1^. Additionally, the La-O-H bending vibration peak, which was originally at 688.9 cm^−1^, shifted to 686.7 cm^−1^ after adsorbing phosphate [56,57]. This indicates that the adsorption of phosphate ions by the nanofibrous composite membrane is a chemical adsorption process. After phosphate ions complex with La^3+^, the electron cloud shifts towards the phosphate group, causing a decrease in the electron cloud density of the La-O-H bond and weakening its strength [58]. This results in a red shift of the vibration frequency. The red shift signifies a decrease in the force constant of the La-O-H bond. This displacement demonstrates a strong complexation between phosphate and La^3+^, achieved through multi-dentate coordination [59].

**Figure 14:**
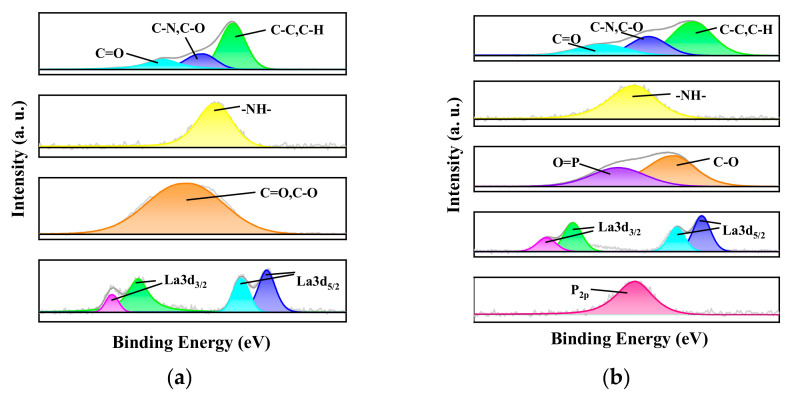
XPS fine spectra: (**a**) XPS fine spectra before adsorption and (**b**) XPS fine spectra after adsorption, respectively.

By analyzing the XPS wide-scan spectra of the membrane before and after adsorption, it is evident that the nanofibrous composite membrane still exhibits a characteristic La 3d peak at 839 eV after adsorption. This indicates that La, incorporated through electrospinning, is firmly embedded in the nanofibrous composite membrane and remains intact. Moreover, a comparison of the XPS spectra of the adsorbed-P membrane and the original membrane reveals a distinct phosphorous characteristic peak at 133 eV for the adsorbed-P membrane [60], confirming the successful adsorption of phosphate ions by the membrane. To further analyze the changes in the elemental states within the membrane, the high-resolution XPS spectra of each element are presented in Figure 14.

After adsorbing phosphate, the nanofibrous composite membrane exhibits a double-peak characteristic of O, with a P=O characteristic peak at 532.75 eV and a C-O characteristic peak at 531.1 eV [61,62]. By comparing the O1s peak areas before and after adsorption, it is evident that the O1s peak after adsorption increases to 1.43 times its original value, further proving the introduction of new oxygen-containing groups in the membrane. Combined with the previous XPS analysis of the nanofibrous composite membrane, it is known that the La characteristic peak shifts positively by 0.5 eV after phosphate adsorption. This indicates the formation of a strong coordination bond (La-O-P) between the phosphate group and La^3+^, leading to a decrease in the electron cloud density of La and consequently shifting the binding energy of the La 3d orbital towards higher binding energy, which is consistent with the observed shift [63].

**Figure 15:**
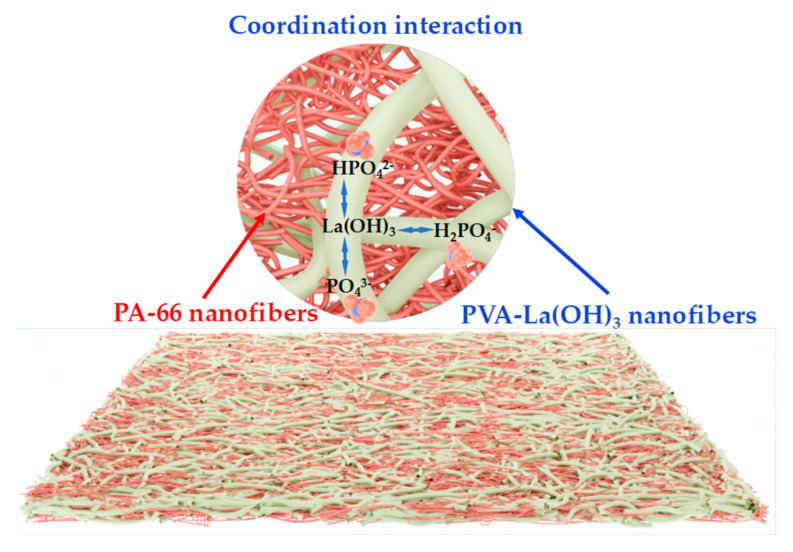
A schematic representation of the interaction between the PA-66/PVA-15%La(OH)_3_ composite membrane and phosphate ions.

A schematic representation, Figure 15, illustrating the adsorption mechanism of the composite membrane toward phosphate ions has been proposed.

The interaction between the La(OH)_3_ component and the phosphate ions are coordination interaction based on ligand exchange reaction [64].

### 3.4. Phosphorus-Release of the Phosphate-Loaded Nanofibrous Composite Membrane

**Figure 16:**
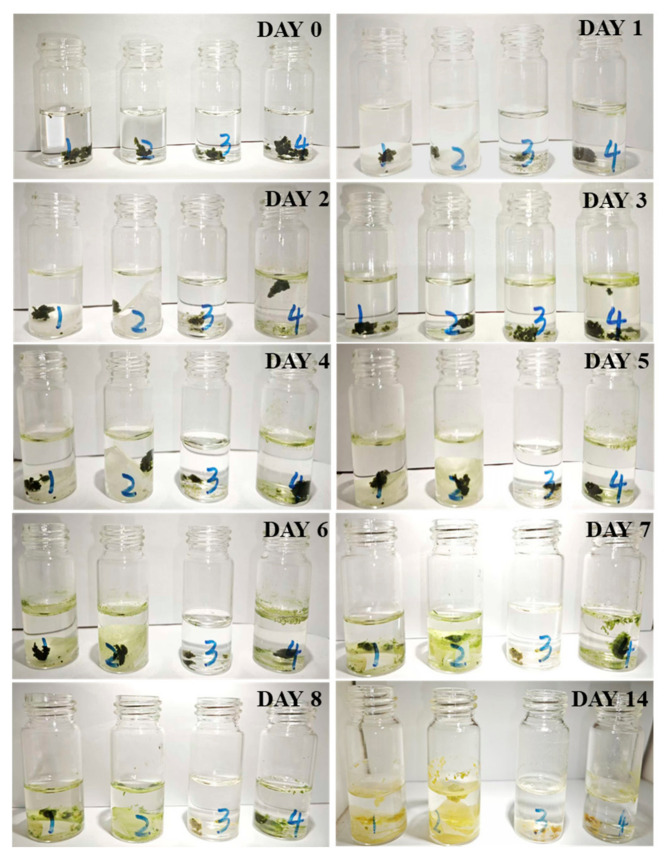
Daily growth status of Mougeotia.

**Figure 17:**
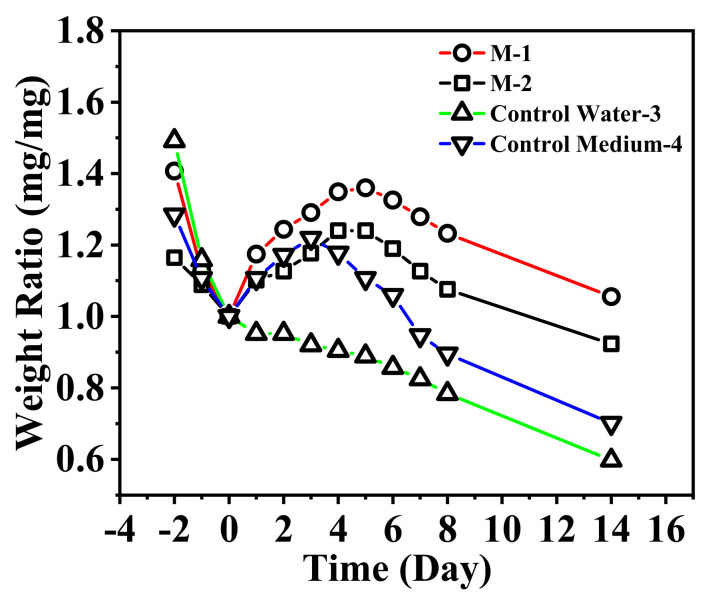
Daily relative mass change of Mougeotia.

The utilization of phosphate after adsorption by the nanofibrous composite membrane was demonstrated by culture of Mougeotia [65], as exhibited in Figure 16 and Figure 17.

The first three days represent the change in Mougeotia mass in the dark, while days 0–14 represent the change under light cultivation. The data confirm that in bottles 1 and 2, Mougeotia reached their mass peaks on the fifth day at 1.36 and 1.24, respectively, after which the mass began to decline. The growth rate of Mougeotia in bottle 1 was higher than that in bottle 2, likely due to the higher amount of phosphate-releasing nanofibrous membrane added to bottle 1, which released more phosphorus fertilizer than bottle 2.

Furthermore, bottle 4, as the control group, reached its mass peak of 1.22 on the third day due to the direct addition of phosphate fertilizer, after which the mass began to decline. Meanwhile, bottle 3, as the blank control group, showed a continuous decline in Mougeotia mass due to the lack of essential nutrients required for its life activities.

By comparing bottles 1, 2, 4, and 3, it can be concluded that phosphate is an essential nutrient for Mougeotia growth. The comparison of bottles 1, 2, and 4 further indicates that the nanofibrous composite membrane can effectively prolong the usability of phosphate fertilizer, demonstrating a significant controlled-release effect.

In summary, the phosphorus elements carried by the nanofibrous composite membrane can be gradually released into the solution over time. This highlights its potential in the field of phosphate controlled-release, enabling the recycling and reuse of phosphorus elements.

### 3.5. Desorption and Regeneration

**Figure 18:**
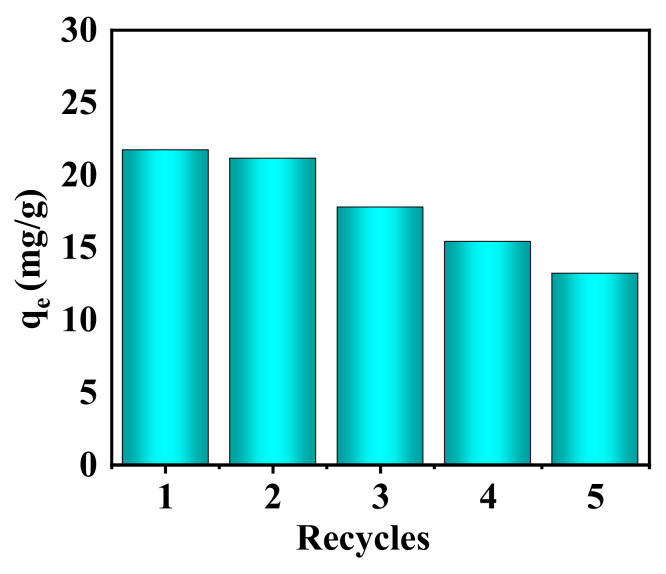
Desorption and regeneration of the PA-66/PVA-15%La(OH)_3_ membrane.

After phosphate adsorption, the nanofibrous composite membrane was desorbed using a 0.5 mol/L NaOH solution, followed by a subsequent adsorption cycle [66]. The results of desorption and regeneration are presented in Figure 18.

As shown in Figure 18, the composite nanofibrous membrane retained 60.7% of its adsorption capacity after five cycles in the phosphate recycling experiment, indicating its significant potential for sustainable use.

## 4. Conclusions

A nanofibrous composite membrane with an interpenetrating structure and selective phosphate ion adsorption capacity was successfully fabricated. In addition to various surface and chemical structure characterizations of the membrane, the effects of different environmental factors on the adsorption performance of the nanofibrous composite membrane were also investigated. The results indicated that the optimal adsorption effect for phosphate ions occurred at pH 7, with an adsorption equilibrium time of 120 min. According to Langmuir model fitting, the maximum adsorption capacity of the nanofibrous composite membrane for phosphate ions was 21.39 mg/g. The membrane also demonstrated good selectivity towards phosphate ions. In dynamic adsorption experiments using membrane modules, the nanofibrous composite membrane could treat 1.74 L of phosphate-containing wastewater at a low flow rate of 1.0 mL/min and 1.46 L at a high flow rate of 2.0 mL/min while maintaining a removal efficiency exceeding 90%, demonstrating good potential for practical application. The adsorption mechanism, as explored via FTIR and XPS, revealed that the phosphate adsorption by the nanofibrous composite membrane is a chemical process involving coordination and complexation. In the Mougeotia culture experiment, the phosphate-loaded nanofibrous composite membrane exhibited a slow-release phosphate fertilizer function. This was beneficial for improving the comprehensive utilization efficiency of phosphate fertilizer, prolonging its effective duration, and achieving the recycling and reuse of phosphorus elements.

## Figures and Tables

**Table 1:** Mougeotia culture protocol.

Constituencies	Group Number	Membrane Weight (mg)	Whether or Not to Add Culture Medium
Experimental group	1	20	Yes
	2	10	Yes
Blank control group	3	0	No
Control group	4	0	Yes

**Table 2:** Fiber diameter and mean pore size of nanofiber membranes.

Samples	Fiber Diameter (μm)		Mean Pore Size (μm)	
	PA-66	PVA	Before Crosslinking	After Crosslinking
PA-66	0.110 ± 0.006	--	0.28	--
PA-66/PVA-5%La(OH)_3_	0.110 ± 0.005	0.565 ± 0.015	0.38	0.35
PA-66/PVA-15%La(OH)_3_	0.109 ± 0.006	0.605 ± 0.012	0.50	0.45

**Table 3:** Pseudo-first-order and pseudo-second-order kinetic fitting parameters.

Kinetic Equations	Parameter	P
$\ln\;(q_{e}-{\;q}_{t})=\;\ln q_{e}-\;k_{1}t$	q_e1_	3.53
	K_1_	0.00657
	R_1_^2^	0.680
$\frac{t}{q_{t}}=\frac{1}{k_{2}q_{e}^{2}}+\frac{1}{q_{e}}t$	q_e2_	9.51
	K_2_ R_2_^2^	0.0108 0.999

**Table 7:** Dynamic snapping model parameters.

Models	Parameter	Low Flow Rate	High Flow Rate
Yoon–Nelson $\frac{C_{t}}{C_{0}}=\frac{1}{1\;+\;\exp\left(k_{\mathrm{YN}}\left(\tau \;-\;t\right)\right)}$	K_YN_ (1/min)	−0.00109	−0.00185
	τ (min)	2008.52	3161
	R^2^	0.92	0.96
Adams–Bohart $\ln\frac{C_{t}}{C_{0}}=k_{\mathrm{AB}}C_{0}t\;-\;k_{\mathrm{AB}}\frac{Z}{U}N_{0}$	K_AB_ (L/(mg·min)	0.000298	0.00098
	N_0_ (mg/L)	41,124	6900
	R^2^	0.71	0.78

K is the model rate constant, “τ” is the 50% penetration time, and N_0_ is the adsorption capacity.

**Table 4:** Adsorption isotherm parameters of the PA-66/PVA-15%La(OH)_3_ membrane for phosphate ions.

Adsorption Isotherms	Isotherm Parameters	P
Langmuir $q_{e}=\frac{q_{m}k_{L}C_{e}}{1+{\;k}_{L}C_{e}}$	K_L_ (L/mg)	0.027
	q_m_ (mg/g)	21.39
	R_L_^2^	0.95
Freundlich $q_{e}=K_{F}C_{e}^{1/n}$	K_F_ (mg/g)/(ppm)1/n	1.52
	1/n	0.52
	R_F_^2^	0.98
Tempkin $q_{e}=\frac{\mathrm{RT}}{b_{T}}\ln(A_{T}C_{e})$	A_T_	0.29
	b_T_	547.82
	R_T_^2^	0.95

**Table 5:** The adsorption capacity of different adsorbents for phosphate ions.

Adsorbents	q_m_ (mg/g)
Chitosan [41]	23.98
Fe/La@BC [42]	44.12
CHM [43]	22.25
Mg–Al–La ternary (hydr)oxides [44]	80.8
La(OH)_3_-modified exfoliated vermiculites [45]	79.6
MCH-La(OH)_3_ [46]	90.2
La-BC [47]	46.37
La_2_O_3_ [48]	17.2
La-P1 [49]	58.2
La-A [49]	44.0
La-CLP [49]	24.6
This work	21.39

**Table 6:** Thermodynamic data of the membrane adsorption for phosphate ions.

T (K)	ΔS^0^ (J/(mol·K))	ΔH^0^ (kJ/mol)	ΔG (kJ/mol)
298	54.44	15.297	−0.93
313			−1.74
323			−2.29
333			−2.83

## Data Availability

The original contributions presented in this study are included in the article. Further inquiries can be directed to the corresponding author.
